# Supplementary material for: Validation of the CLIF-C OF Score and CLIF-C ACLF Score to Predict Transplant-Free Survival in Patients with Liver Cirrhosis and Concomitant Need for Intensive Care Unit Treatment
Source: Medicina (Kaunas). 2023 Apr 29;59(5):866. doi: 10.3390/medicina59050866 (PMC10221777; doi:10.3390/medicina59050866)
Supplement: Supplementary file 1 [file medicina-59-00866-s001.zip › medicina-2313605-supplementary.pdf]

Table S1. Analyses of potential predictors for the composite endpoint of death or need for liver transplantation within 28-days, 90-days or 365-days after ICU admission in the entire cohort of patients with concomitant need for intensive care treatment using multiple logistic regression models. Only significant variables in univariable logistic regression analyses were included into the models. Not significant in univariable analyses were age, infection at ICU admission and sodium. Bold values indicate level of significance ($p < 0.05$). Abbreviations: OD: odds ratio; CLIF-C: chronic liver failure consortium; OF: organ failure; CRP: C-reactive protein; WBC: white blood cells.

	**Model 1 28-days survival**		**Model 2 90-days survival**		**Model 3 365-days survival**	
	**OR**	**p-value**	**OR**	**p-value**	**OR**	**p-value**
CLIF-C OF score	1.305 (1.115 – 1.527)	**0.001**	1.336 (1.106 – 1.615)	**0.003**	1.283 (1.055 – 1.561)	**0.012**
platelets	0.996 (0.992 – 1.001)	0.142	0.995 (0.989 – 1.000)	0.051	0.999 (0.993 – 1.004)	0.631
CRP	1.007 (1.001 – 1.013)	**0.034**	1.005 (0.998 – 1.013)	0.186	1.001 (0.994 – 1.008)	0.748
WBC	1.004 (0.951 – 1.061)	0.876	1.014 (0.946 – 1.086)	0.699	1.007 (0.938 – 1.081)	0.854

Table S2. Analyses of potential predictors for the composite endpoint of death or need for liver transplantation within 28-days, 90-days or 365-days after ICU admission in the subgroup of patients with ACLF and concomitant need for intensive care support using multiple logistic regression models. White blood cells (WBC) was not included due to collinearity with CLIF-C ACLF score. Only significant variables in univariable logistic regression analyses were included into the models. Not significant in univariable analyses were age, infection at ICU admission and sodium. Bold values indicate level of significance ($p < 0.05$). Abbreviations: OR: odds ratio; CLIF-C: chronic liver failure consortium; ACLF: acute-on-chronic liver failure; CRP: C-reactive protein.

	**Model 1 28-days survival**		**Model 2 90-days survival**		**Model 3 365-days survival**	
	**OR**	**p-value**	**OR**	**p-value**	**OR**	**p-value**
CLIF-C ACLF score	1.095 (1.041 – 1.151)	**<0.001**	1.092 (1.025 – 1.164)	**0.007**	1.079 (1.009 – 1.154)	**0.027**
Platelets	0.995 (0.990 – 1.001)	0.081	0.994 (0.998 – 1.000)	0.051	0.997 (0.991 – 1.003)	0.349
CRP	1.005 (0.997 – 1.013)	0.198	1.001 (0.992 – 1.010)	0.859	0.997 (0.989 – 1.006)	0.516
